# Positron emission tomography: Evolving modalities, radiopharmaceuticals and professional collaboration

**DOI:** 10.1002/jmrs.629

**Published:** 2022-11-05

**Authors:** Sally L. Ayesa, Andrew Murphy

**Affiliations:** ^1^ Department of Medical Imaging & Nuclear Medicine Gosford & Wyong Hospitals Gosford New South Wales Australia; ^2^ Department of Nuclear Medicine Royal North Shore Hospital St Leonards New South Wales Australia; ^3^ School of Medicine University of Sydney Campderdown New South Wales Australia; ^4^ Department of Medical Imaging Princess Alexandra Hospital Woolloongabba QLD Australia; ^5^ School of Clinical Sciences Faculty of Health Queensland University of Technology Brisbane QLD Australia

## Abstract

Positron emission tomography (PET) as an imaging modality has undergone considerable innovation over the past few decades. Hybrid anatomical and functional imaging has become commonplace (initially PET/CT but now also PET/MRI) with improvements in imaging technology continually delivering studies with increasing diagnostic accuracy and decreasing radiation dose to the patient. More and more radiopharmaceuticals have emerged from the research sphere into clinical practice, with the traditional PET workhorse tracer ^18^F‐FDG accompanied by a range of novel radiopharmaceuticals with specific molecular targets. Imaging facilities offering PET/CT and PET/MRI provide a unique collaborative environment for the medical imaging multidisciplinary team. Diagnostic radiographers and nuclear medicine technologists especially have the opportunity to not only work together but also share knowledge and technical skills, ultimately benefitting the quality of patient care.

## Graphical abstract

PET has experienced exponential growth over the last few decades in terms of innovative technologies and novel radiopharmaceuticals. This has fostered increasing collaboration between radiographers and nuclear medicine technologists.

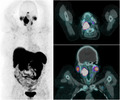

## Background

Radiology and nuclear medicine are unique fields within health care, interconnected with technology and often at the forefront of scientific innovation and evolution. Indeed, for PET, the last 30 years have witnessed a meteoric rise in clinical applications. Positrons were first theorised and observed in the early 1930s, eventually leading to the development of the first human research PET scanner by the 1970s.^1^ By April 1991, the Journal of Nuclear Medicine proudly asserted that the time had come to move PET scanning from the realm of research into mainstream health care.^2^ Initially developed as an imaging modality for assessment of the brain, by the end of the 1990s the utility of ^18^F‐FDG (fluorodeoxyglucose) PET for oncology patients was starting to be utilised in clinical practice.

In the early clinical days of PET scanning, as it remains to this day, the radiopharmaceutical ^18^F‐FDG (a glucose analogue) was central. In the form of FDG was an elegant substitute for mapping the metabolic activity of the body, demonstrating how much energy the different tissues were consuming at the time the patient was scanned. Cells with higher metabolic activity, a common but not universal characteristic of high‐grade malignancy, would accumulate more FDG, and by way of a positron‐emitting ^18^F atom tagged into the molecule, these cells would emit the largest number of photons. With the development of the PET scanner, we could now see where in the body those cells were. For so many patients around the world, this was revolutionary.

## Evolving Modalities

Isolated PET scanning has been all but completely superseded by the hybrid imaging technique of PET/CT, fusing the functional PET data and anatomical CT data. The superior attenuation correction and honed ability to localise the structure accumulating FDG to a specific tissue location meant that reports were issued with superior diagnostic accuracy. The developments of time‐of‐flight technology and digital scanners, as well as advances in tomographic reconstruction algorithms, have further improved spatial and contrast resolution. Most recently, total body PET/CT has started to be used more frequently in clinical practice across Australia and New Zealand providing a long‐axial scan range which allows PET data to be obtained in a single bed position acquisition. Total body PET/CT scanners allow for lower administered activity, reduced radiation dose to the patient, shortened scan times and superior image quality by way of improved signal‐to‐noise ratios.^3^

Currently, PET/MRI is in a time of flux as it emerges from the realm of research and into clinical practice in Australia and around the world. With MRI advantageous due to its superior soft tissue contrast and lack of ionising radiation, PET/MRI has found utility in paediatric patients, in the assessment of tumours in areas traditionally imaged with MRI such as the brain and hepatobiliary system, and non‐oncological applications such as assessment of inflammatory bowel disease, neurodegenerative disorders and the heart.^4^

These hybrid imaging modalities have provided an opportunity for nuclear medicine technologists and diagnostic radiographers to work together in improving the diagnosis and monitoring of various conditions. Diagnostic radiographers tend to work in fast‐paced environments in which patient contact is limited to the brief period before and after an examination. Exam times are not strict, and bookings can be quickly rescheduled make way for high‐acuity studies. In contrast, PET imaging relies heavily on regimented timing schedules accounting for radiopharmaceutical half‐life and availability. This requires the multidisciplinary imaging team to act in sync.

## Evolving Radiopharmaceuticals

Whilst ^18^F‐FDG continues to stand tall as the workhorse radiopharmaceutical of PET imaging, in recent decades the number of radiopharmaceuticals available in the research and clinical space has exploded. There are targeted tracers to assess everything from amyloid plaque deposition in Alzheimer's disease, to oestrogen receptor expression in breast cancer patients and for characterisation of primary brain tumours.

Building on discoveries in the late 1980s and 1990s, radiopharmaceuticals targeting prostate‐specific membrane antigen (PSMA) were developed through the early 2000s. PSMA is physiologically expressed in the prostate and several other organs in the body, however, its high rate of expression of primary prostate cancer and nodal disease (approximately 95%) drove its development as a specific tracer for assessment and treatment of prostate cancer.^5^ Two radiopharmaceuticals emerged for PSMA PET imaging (^68^Ga‐PSMA‐11 and ^18^F‐DCFPyL) which served important clinical utility for staging, treatment monitoring and radiotherapy planning, with PSMA PET/CT shown to have high diagnostic utility compared with some conventional imaging methods such as bone scan and diagnostic CT.^6^

Neuroendocrine tumours are represented in a small but unique subset of oncology patients. Targeted imaging in this group is not new, with the single‐photon emitting ^111^In‐Octreotide exploiting the propensity of these tumours to express somatostatin receptors. With these tumours often being well‐differentiated and slow growing, ^18^F‐FDG PET/CT was of limited utility. In addition, there were inherent challenges with ^111^In‐octreotide in terms of spatial resolution, prolonged duration of the study and radiation dose. The clinical emergence of ^68^Ga‐DOTATATE in the early 2010s offered an alternative radiopharmaceutical that boasted superior lesion to background uptake, creating a more sensitive and diagnostically accurate test.^7^

In theranostics, a positron‐emitting radioactive isotope is first paired with a specific pharmaceutical to allow diagnostic imaging targeted at a molecular level. A second isotope emitting higher energy beta or alpha particles can then be paired to the same pharmaceutical to deliver targeted treatment to the affected cells. With favourable physiological biodistribution and the potential to form a theranostic pair by swapping out the positron‐emitting ^68^Ga with the beta‐emitting ^177^Lu, PSMA and DOTATATE pharmaceuticals soon found their way into the therapy space. Patients with previously difficult‐to‐target metastatic malignancies now had an option in which local radiotherapy could be delivered directly to the cellular level by exploiting the characteristics of the tumour cells themselves. Trials of ^177^Lu‐PSMA and ^177^Lu‐DOTATATE have demonstrated promising results compared to conventional systemic therapy for selected groups of patients with metastatic prostate and neuroendocrine cancers.^8^, ^9^

As we write, exciting new pharmaceuticals such as those which act as fibroblast‐activation‐protein inhibitors (FAPIs) are being trialled all over the world, on the cusp of widespread clinical implementation. Images from ^68^Ga‐FAPI PET/CT graced the cover of the Journal of Nuclear Medicine in 2019, exquisitely showcasing its utility in assessment of a broader range of oncology diagnoses.^10^ Excitingly, the normal physiological distribution of the tracer did not include the brain (as ^18^F‐FDG does) opening the possibility of developing FAPI as a targeted therapy for a broad range of deadly cancers.

Two articles within this issue showcase the value that PET scans have the potential to add to our patients, both in terms of innovative applications of traditional radiopharmaceuticals and the potential value of novel agents.

The importance of ^18^F‐FDG in the assessment of colorectal cancer patients has been long held, with tumour uptake traditionally assessed with visual assessment and semi‐quantitative measures such as SUVmax (maximum specific uptake value). Smith and colleagues present data from their pilot project, where they have considered quantitative methods for evaluating the metabolic activity within treated lesions and thereby considering the shortest progress imaging interval by which eventual response to treatment can be predicted.^11^ If a chemoradiotherapy regime can be considered successful (or unsuccessful) at an earlier time point, there is potential to tailor and adapt treatment regimens to improve patient outcomes.

The second article explores the role which ^68^Ga‐nitroimidazole PET/CT can play in patients with difficult‐to‐treat dormant tuberculosis.^12^ Bresser and colleagues present a series of cases where ^68^Ga‐nitroimidazole PET/CT measured the hypoxic uptake within tuberculosis lesions, considering how lesion characteristics may influence delivery of treatment agents and thereby success of the regime. This represents just one of so many novel PET tracers being applied to the clinical landscape with the potential to impact how we diagnose and monitor patients by modelling disease processes on a cellular level.

## Professional Collaboration

The collaboration with nuclear medicine technologists and medical staff presents diagnostic radiographers with a new experience in a highly functioning, multifaceted team that is still quite young relative to the field of medicine overall. Opportunities for research in collaboration with academics, whilst being able to give meaningful guidance around radiation safety, imaging protocols and quality improvement, will offer job satisfaction and a change of scenery to the conventional radiography modalities.^13^

Combining CT or MRI with PET imaging has offered diagnostic radiographers and nuclear medicine technologists new avenues to utilise their clinical and technical expertise within the changing diagnostic and therapeutic landscape of nuclear medicine. The two professions have worked in parallel for so long – to work together in such a new and exciting field of imaging is a unique opportunity. PET/CT or PET/MR imaging is a form of precision medicine where clinical skills, efficiency and technical knowhow are only as good as one's ability to converse and empathise with patients. Radiographers are given the opportunity to learn from nuclear medicine colleagues the nuances of patient care and professionalism whilst balancing the time‐critical nature of radiopharmaceuticals. Conversely, nuclear medicine technologists can learn and appreciate the complexities of operating diagnostic computed tomography and magnetic resonance scanners.^14^ As radiographers must have a thorough understanding of cross‐sectional anatomy and possess skills to recognise medically urgent imaging diagnoses, radiographers are able to share this knowledge and experience with their nuclear medicine colleagues.^15^

Moving forward, training standards and agreement on competency for both professions will be important to ensure this partnership between teams to deliver precision medicine does not fold under its own complexity and disagreements around ownership of roles.^16^ There is the potential to improve collaborative knowledge of radiation science, image acquisition, technical challenges and clinical problem‐solving, ultimately benefiting some of healthcare's most vulnerable patients.

## Conflict of Interest

The authors do not have any conflicts to declare.
